# Rectal bleeding as a symptom of solitary rectal ulcer syndrome mimicking rectal neoplasm on colonoscopy: A case report

**DOI:** 10.1002/ccr3.7277

**Published:** 2023-04-24

**Authors:** Masood Faghih Dinevari, Amirtaher Eftekharsadat, Mahdi Tarverdizadeh, Seyyed Mahdi Rasulimanesh, Ali Riazi, Amirreza Jabbaripour Sarmadian, Samaneh Abbasian

**Affiliations:** ^1^ Liver and Gastrointestinal Diseases Research Center Tabriz University of Medical Sciences Tabriz Iran; ^2^ Department of Pathology Tabriz University of Medical Sciences Tabriz Iran

**Keywords:** gastrointestinal hemorrhage, hematochezia, rectal neoplasm, constipation

## Abstract

Patients complaining of rectal bleeding, constipation, and a suspicious mass in colonoscopy should undergo biopsy. Histological features such as fibromuscular obliteration in the lamina propria favor SRUS, a benign disorder.

## Abstract 2

Solitary rectal ulcer syndrome (SRUS) is a rare benign rectal disorder often misdiagnosed with other diseases, such as IBD, constipation, and malignancies. Therefore, it is necessary to know its diagnostic features and treatment. In this report, we present a case of a polypoid solitary rectal ulcer presented with rectal bleeding and chronic constipation mimicking rectal neoplasm on colonoscopy. Histopathological study showed the characteristics of SRUS, the most important of which was fibromuscular obliteration in the lamina propria.

## 1 INTRODUCTION

Solitary rectal ulcer syndrome (SRUS) is a rare benign rectal disorder, with an estimated prevalence of one in 100,000 individuals annually.^1^, ^2^, ^3^ Its prevalence does not differ in gender and can occur at any age, mainly in the third decade of men's lives and the fourth decade of women's.^3^ Despite its name, it is not limited to ulcers and can affect different parts of the rectum and gastrointestinal tract, so some patients do not even have ulcers.^2^, ^3^

It is characterized by straining and painful defecation, a sense of incomplete evacuation, perineal pain, tenesmus, chronic constipation, mucus discharge, fresh lower gastrointestinal bleeding, and rarely rectal prolapse; however, some patients may be asymptomatic.^1^, ^2^, ^3^, ^4^ The pathogenesis and etiology of SRUS are not well understood; several factors may be involved; the most critically discussed factors include direct trauma and causes of local ischemia such as straining, self‐induced trauma, intussusception, rectal prolapse, anatomical anomalies, and paradoxical contraction of the pelvic floor muscles.^2^, ^3^, ^5^

Due to its rare occurrence, it is not adequately diagnosed and treated, which makes it often misdiagnosed with other diseases such as inflammatory bowel disease (IBD), constipation, and malignancies. Therefore, it is necessary to know its diagnostic features and treatment.^1^, ^2^, ^3^, ^4^ Diagnosis is mainly based on clinical symptoms, endoscopic, histopathologic, and imaging findings, which must be confirmed with histology findings to avoid misdiagnosis.^1^, ^2^ In general, the treatment is based on the severity of the disease and the patient's symptoms, such as the severity of bleeding, constipation, ulcers, and rectal prolapse. Treatment options include conservative treatment, medical therapy, lifestyle changes, biofeedback therapy, and surgery.^1^, ^2^, ^3^

This report presents a case of a polypoid solitary rectal ulcer in a 19‐year‐old man who came to the gastroenterology clinic with rectal bleeding and chronic constipation, the diagnostic procedures to rule out other critical differential diagnoses, and the treatments performed.

## 2 CASE PRESENTATION

A 19‐year‐old man came to the gastroenterology outpatient clinic complaining of rectal bleeding and severe constipation. He had a history of constipation and tenesmus since childhood. His symptoms intensified, and presented with rectal bleeding recently, so he came to the clinic. He did not have any past medical or past surgical history and did not use any medication. His family history of bowel disease and colorectal cancers were unremarkable. He had no complaint of abdominal pain or recent weight loss.

At First, general physical examinations were performed, and there were no abnormalities except pallor. Digital rectal examination was impossible due to partial obstruction of the rectal lumen. His blood pressure was 115/75 mm Hg; his heart rate was 96 bpm; his body temperature was 36.6°C; his respiratory rate was 18; and his oxygen saturation was 97% without supplementation.

On his laboratory investigation, his white blood cell count was 7600; his hemoglobin was 10.9 gm/dl; his hematocrit was 38.8%; his mean corpuscular volume was 65 fl; his ferritin was 6.3 ng/mL; his platelet count was 361,000; and his erythrocyte sedimentation rate was 5 mm/h. These findings indicated hypochromic microcytic anemia, probably due to iron deficiency anemia. Coagulation profile and liver function tests were within normal ranges. Examinations of stool for parasites and cultures were negative.

**FIGURE 1:**
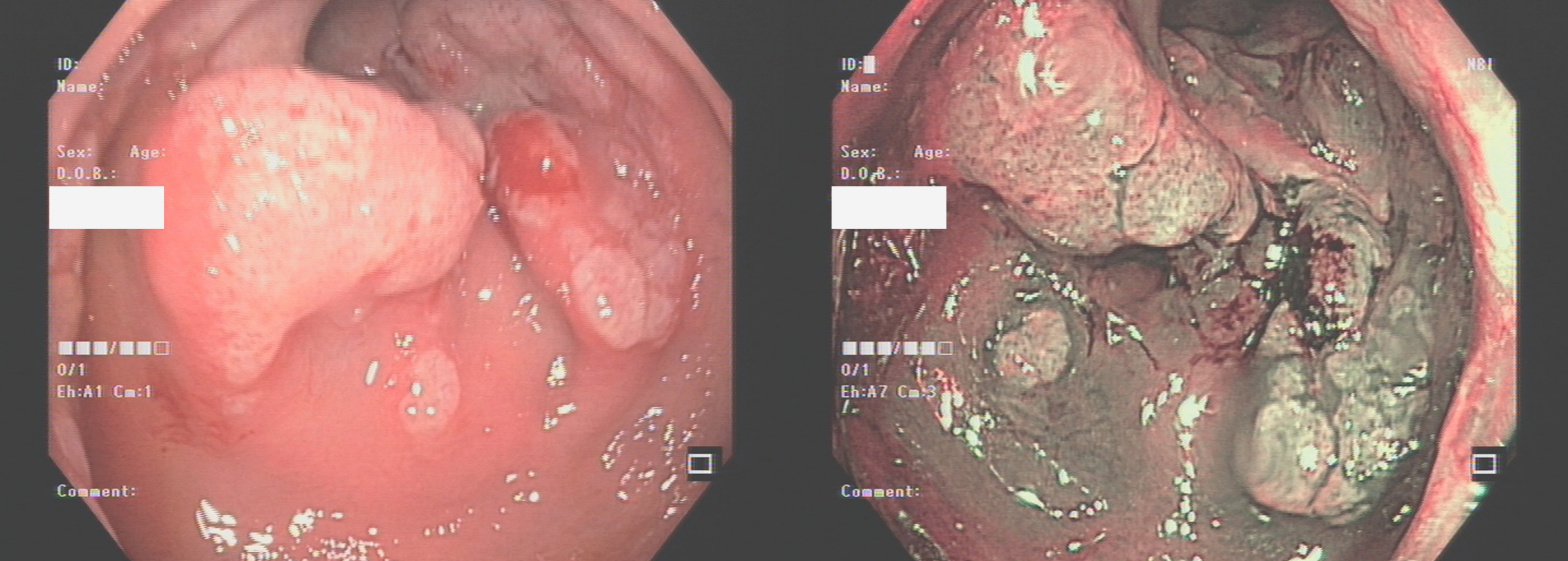
Two sections of the colonoscopy findings of the patient showing a large semi‐circumferential infiltrative mass.

Colonoscopy was performed according to the indications in this patient, such as rectal bleeding and chronic constipation. It revealed one large semi‐circumferential infiltrative mass lesion with a polypoid appearance in the rectum within 5 cm of the anal verge, which was suspected to be malignant, so biopsy was taken (Figure 1). The entire colon was observed up to the cecum, which was normal.

**FIGURE 2:**
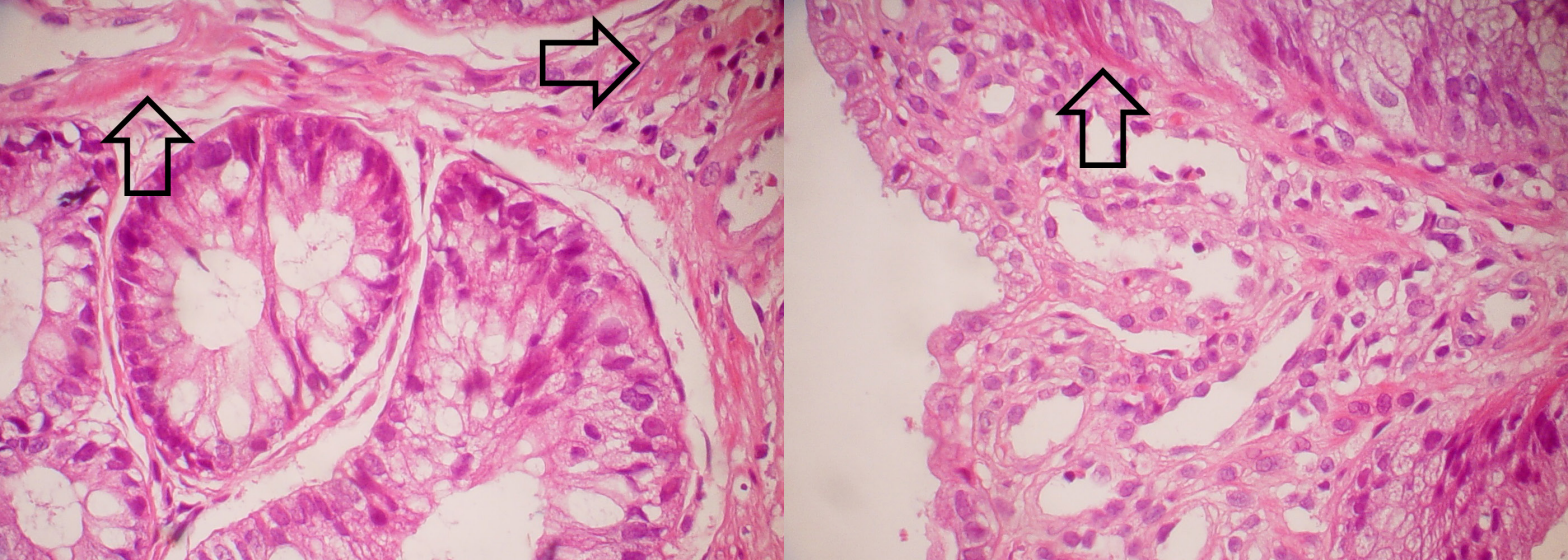
Two sections of the histopathological findings of the patient showing features of SRUS (Fibromuscular obliteration shown with arrow).

Histopathological study revealed features of solitary rectal ulcer, including fibrosis and extension of smooth muscle fibers in the lamina propria with diamond‐shaped crypts, crypt regenerative hyperplasia, surface erosion, crypt hyperplasia, ecstatic capillaries, and minimal inflammation (Figure 2). These histologic features and colonoscopy findings were suggestive of polypoid solitary rectal ulcer.

Treatment with bulking agents, hydrocortisone, and mesalamine enemas was prescribed, and he was advised to have a high‐fiber diet. During the follow‐up visits, his symptoms, such as constipation and hematochezia, were alleviated. Now he is under the care of a gastroenterologist and advised to present regularly to perform follow‐up with colonoscopy examination.

## 3 DISCUSSION

SRUS is a spectrum of clinicopathological abnormalities^4^; in this report, we presented a polypoid lesion of the rectum, a rare variant of SRUS.^6^, ^7^ As mentioned, the exact pathogenesis of SRUS is unknown, and several factors are proposed to play roles in its development.^5^ In our case, it was probably due to excessive straining to defecate, as a result of chronic constipation.

The most common symptoms of SRUS are rectal bleeding, constipation, anemia, and abdominal pain.^7^ In our case, rectal bleeding, chronic constipation, and mild anemia were present, but there was no abdominal pain, and the abdominal examination was normal. The most common colonoscopy findings are solitary lesions, ulcerative lesions, multiple lesions, and erythematous lesions^7^; however, some patients do not have any ulcers.^1^, ^2^ Ulcers are usually located in the anterolateral wall of the rectum within 3–10 cm of the anal verge.^1^, ^2^, ^7^ In addition, prolapse of rectal mucosa may be seen in a variety of situations such as the margin of hemorrhoid, diverticula, and any polypoid lesion. The partial mucosal prolapse of rectal mucosa (Not full‐thickness prolapse) in the anterior wall of the rectum is more favorable for SRUS.^8^ In our case, there was a solitary large semi‐circumferential infiltrative mass lesion with a polypoid appearance within 5 cm of the anal verge.

The polypoid appearance of this lesion may be misdiagnosed with an inflammatory polyp, hyperplastic polyp, or rectal carcinoma leading to delayed diagnosis and treatment, which can cause several complications, so it is necessary to have biopsies for a definite diagnosis.^6^ Histopathological findings are the gold standard for the definite diagnosis of SRUS. Histologic features are fibromuscular obliteration in the lamina propria, extension of muscle fiber upward between crypts, thickening of the mucosal layer with crypts distortion, mucosal cell proliferation with hyperplastic, and serrated mucosa, mucosal glands distortion, glandular crypt abnormalities, surface erosion, diffuse collagen deposition in lamina propria, mild inflammation, and reactive epithelial atypia.^3^, ^7^, ^9^

In our case, histologic features were fibrosis and extension of smooth muscle fibers in the lamina propria with diamond‐shaped crypts, crypt regenerative hyperplasia, surface erosion, and minimal inflammation. In order to distinguish SRUS from dysplasia, minimal inflammation and crypt regenerative hyperplasia are important features. However, crypt regenerative hyperplasia along with extension of smooth muscle fibers in the lamina propria around the crypts, may lead to a misdiagnosis of invasive adenocarcinoma, so an expert gastrointestinal pathologist should be consulted to confirm the diagnosis.

In order to distinguish SRUS from IBD, there are two important histologic features, the presence of smooth muscle between crypts and ulceration without the infiltration of chronic inflammatory cells. Furthermore, the most critical differential diagnosis is rectal carcinoma; which may have similar appearance to SRUS on colonoscopy, such as thickened mucosa and strictures; so, histological findings are critical, especially in patients over 40 years of age. The differential features of SRUS from rectal cancer are summarized in Table 1.^7^, ^8^, ^9^, ^10^

**TABLE 1:** Summary of the differential features of SRUS from rectal cancer.

Common Features	Solitary rectal ulcer	Rectal cancer
Age	Any age	Older ages
Symptoms	Rectal bleeding Chronic constipation	Rectal bleeding Changes in bowel habits Weight loss
Lesion Location	Lower rectum	Lower rectum
Laboratory Findings	Iron deficiency anemia	Iron deficiency anemia
Endoscopic findings	Solitary or Multiple ulceration	Exophytic endoluminal masses Polypoid or Nonpolypoid ulcerated lesions
Histologic findings	Fibromuscular obliteration in the lamina propria Extension of muscle fibers upwards between crypts Diffuse collagen deposition in the lamina propria Glandular crypt abnormalities Mild inflammation	Well or poorly differentiated adenocarcinoma Cellular atypia Pleomorphism

Treatment is based on the severity of the disease and the patient's symptoms, such as the severity of bleeding, constipation, ulcers, and rectal prolapse. Patient education and lifestyle changes are the first steps of treatment.^2^ In the next step, conservative therapy is suggested. Surgery is suggested for patients who do not respond to conservative therapy or who have rectal prolapse. Conservative therapy includes a high‐fiber diet, intermittent use of laxatives, corticosteroids, sulfasalazine, and 5‐aminosalicylate enemas. Procedures performed for SRUS include rectopexy, Delorme's procedure, local excision, and perineal proctectomy.^2^, ^3^, ^11^ Our patient was treated with a combination of lifestyle changes and conservative therapy that relieved his symptoms.

In conclusion, for patients with complaints of rectal bleeding with constipation and the presence of a suspicious mass in colonoscopy, a biopsy should be taken to decide on a treatment plan, because it differs based on the nature of the mass. Histological features such as fibromuscular obliteration in the lamina propria is in favor of SRUS; however, IBD and malignancies should be ruled out.

## AUTHOR CONTRIBUTIONS

**Masood Faghih Dinevari:** Conceptualization; methodology; project administration; supervision. **Amirtaher Eftekharsadat:** Conceptualization; methodology; supervision. **Mahdi Tarverdizadeh:** Data curation; investigation; validation. **Seyyed Mahdi Rasulimanesh:** Data curation; investigation; validation. **Ali Riazi:** Data curation; validation; writing – original draft. **Amirreza Jabbaripour Sarmadian:** Writing – original draft; writing – review and editing. **Samaneh Abbasian:** Data curation; project administration; writing – original draft; writing – review and editing.

## FUNDING INFORMATION

There was no financial support or funding for this research.

## CONFLICT OF INTEREST STATEMENT

The authors declare no financial and non‐financial competing interests related to this work.

## ETHICS STATEMENT

This study was performed according to the principles outlined by the World Medical Association's Declaration of Helsinki on experimentation involving human subjects, as revised in 2000, and was approved by the Tabriz University of Medical Sciences ethics committee with the approval number IR.TBZMED.REC.1399.1101 on 2021/02/22.

## CONSENT STATEMENT

The patient was informed regarding publishing this case report, and written informed consent was obtained to publish this report under the journal's patient consent policy.

## Data Availability

Data are available from the corresponding author on reasonable request.
